# Dosimetric and Radiobiological Comparison of Five Techniques for Postmastectomy Radiotherapy with Simultaneous Integrated Boost

**DOI:** 10.1155/2020/9097352

**Published:** 2020-07-21

**Authors:** Du Tang, Zhan Liang, Fada Guan, Zhen Yang

**Affiliations:** ^1^Department of Oncology, Xiangya Hospital, Central South University, Changsha, Hunan Province, China; ^2^Department of Radiation Physics, Division of Radiation Oncology, The University of Texas MD Anderson Cancer Center, Houston, TX, USA

## Abstract

**Purpose:**

To compare five techniques for the postmastectomy radiotherapy (PMRT) with simultaneous integrated boost (SIB).

**Materials and Methods:**

Twenty patients with left-sided breast cancer were retrospectively selected. Five treatment plans were created for each patient: TomoDirect (TD), unblocked helical TomoTherapy (unb-HT), blocked HT (b-HT), hybrid intensity-modulated radiotherapy (hy-IMRT), and fixed-field IMRT (ff-IMRT). A dose of 50.4 Gy in 28 fractions to PTV_total_ and 60.2 Gy in 28 fractions to PTV_boost_ were prescribed. The dosimetric parameters for targets and organs at risk (OARs), the normal tissue complication probability (NTCP), the second cancer complication probability (SCCP) for OARs, and the treatment efficiency were assessed and compared.

**Results:**

TD plans and hy-IMRT plans had similar good dose coverage and homogeneity for both PTV_boost_ and PTV_total_ and superior dose sparing for the lungs and heart. The ff-IMRT plans had similar dosimetric results for the target volumes compared with the TD and hy-IMRT plans, but gave a relatively higher NTCP and SCCP for the lungs. The unb-HT plans exhibited the highest OAR mean dose, highest NTCP for the lungs (0.97 ± 1.25‰) and heart (4.58 ± 3.62%), and highest SCCP for the lungs (3.57 ± 0.05%) and contralateral breast (2.75 ± 0.29%) among all techniques. The b-HT plans significantly outperformed unb-HT plans with respect to the sparing of the lungs and heart. This technique also showed the best conformity index (0.73 ± 0.08) for PTV_boost_ and the optimal NTCP for the lungs (0.03 ± 0.03‰) and heart (0.61 ± 0.73%). Concerning the delivery efficiency, the hy-IMRT and ff-IMRT achieved much higher delivery efficiency compared with TomoTherapy plans.

**Conclusion:**

Of the five techniques studied, TD and hy-IMRT are considered the preferable options for PMRT with SIB for left-sided breast cancer treatment and can be routinely applied in clinical practice.

## 1. Introduction

Postmastectomy radiotherapy (PMRT) plays a critical role in breast cancer treatment. Previous studies have demonstrated a significant improvement in overall and local survival after PMRT in breast cancer patients [1, 2]. The use of a tumor-bed boost scheme has shown the further improvement in the local-regional control for patients with high-risk features [3]. Although there is lack of randomized data to guide the boost dose setting, the boost technique is accepted as a routine practice in many centers [3–5]. According to our local protocol, the simultaneous integrated boost (SIB) scheme is administrated for high-risk cases. The delivery of PMRT with SIB for patients is challenging due to the large target volume size, high prescription dose, and proximity to critical organs, especially for patients who suffer from left-sided breast cancer. Therefore, it is of great importance to find the optimal technique for PMRT with SIB.

Advanced RT techniques, such as fixed-field IMRT (ff-IMRT), or further developments, such as TomoTherapy, now allow treating multiple target volumes with different prescribed doses in a single plan. Previous studies have demonstrated that IMRT and TomoTherapy outperformed conventional three-dimensional conformal radiotherapy (3DCRT) for the whole breast irradiation [6–8]. Several studies have already been published comparing various treatment modalities for PMRT treatment without the boost scheme [9–11] and the whole breast irradiation [8, 12, 13]. Very few publications, however, have focused on such comparison in PMRT with SIB. Since the dose prescription scheme and the definition of the target volumes in previous studies were different from those for PMRT with SIB, the results in previous studies cannot simply be transferred to the treatment for this group of patients. Dedicated planning studies for PMRT with SIB are therefore necessary to identify the appropriate techniques. Moreover, the feasibility of TomoDirect (TD) [14], a nonrotational treatment option in the new version of TomoTherapy, is yet to be studied for PMRT with SIB. In this study, the comparison of dosimetric parameters, radiobiology indices, and delivery efficiency among the TD, helical TomoTherapy (HT), ff-IMRT, and hy-IMRT plans was performed for PMRT with SIB.

## 2. Materials and Methods

### 2.1. Patient Selection, CT Simulation, and Contouring

Twenty patients who underwent PMRT with SIB in Xiangya Hospital of Central South University between December 2016 and July 2019 were retrospectively selected in this study. The inclusion criteria were as follows: (1) female patients older than 18 years with left-sided breast carcinoma, who had undergone radical mastectomy; (2) diagnosis of invasive cancer was pathologically confirmed; (3) surgical margin was negative; (4) had received chemotherapy in accordance with standards and guidelines before radiotherapy.  Table 1 outlines the patient characteristics.

The free-breathing CT scans were obtained from the level of the mandible to the lower abdomen on a SOMATOM Definition AS CT scanner (Siemens Medical Solutions, Erlangen, Germany) with a slice thickness of 3 mm. Patients were immobilized on a customized evacuated vacuum bag, in the supine position with the use of a wing board for arm positioning above the head. The clinical target volume (CTV) and OARs for each patient were contoured by the attending physician. The CTV was defined as chest wall (CW), internal mammary nodes (IMNs), and axillary and supraclavicular lymph nodes. A margin of 5 mm was added to the CTV, given the PTV_total_, to consider for daily setup and organ motion. The boost volume was drawn to include the site of surgically removed tumor, according to pre- and postoperative CT images and surgical reports. A setup safety margin of 5 mm was added, given the PTV_boost_. Both PTV_total_ and PTV_boost_ were constrained to be inside the body surface. A 5 mm bolus was applied to the skin of the left CW to ensure adequate skin dose [1]. The following OARs were considered: contralateral breast, lungs (including ipsilateral lung and contralateral lung), heart, and spinal cord.

### 2.2. Treatment Planning

6 MV photons were used in all the plans. The prescription doses (*D*_p_) were 50.4 Gy in 28 fractions and 60.2 Gy in 28 fractions for PTV_total_ and PTV_boost_, respectively. The optimization goal was to achieve ≥95% of PTV_total_ and PTV_boost_ receives 100% of *D*_p_. Meanwhile, the following dose constraints were used for OARs in the optimization: (1) *D*_max_ < 52 Gy, *V*_5Gy_ < 45%, *V*_20Gy_ < 18%, *V*_45Gy_ < 5% for the ipsilateral lung; (2) *D*_max_ < 20 Gy, *V*_5Gy_ < 15% for the contralateral lung; (3) *D*_max_ < 50 Gy, *V*_20Gy_ < 10%, *V*_30Gy_ < 7%, *V*_40Gy_ < 3% for the heart; (4) *D*_max_ < 10 Gy, *V*_5Gy_ < 30% for the contralateral breast.

The TD and HT plans were created and optimized on the TomoHD™ planning station (version 5.1.1.6, Accuray Inc., Sunnyvale, CA). For TD plans, the paired tangential beam angles were used. Moreover, two additional fields with modified gantry angles of ±10° from the tangential beam set and an anterior supraclavicular field were employed. Two HT plans were created with and without a complete blocking structure. For HT plans with the blocking structure (b-HT), the primary radiation beams were limited to pass through part of the lungs and heart to improve the dose sparing. For HT plans without the blocking structure (unb-HT), the primary beams can pass through the lungs and heart. A jaw size of 2.5 cm, a pitch of 0.25, a modulation factor (MF) of 2.0, and a fine calculation grid of 1.95 × 1.95 mm^2^ were set for all TomoTherapy plans. The dose calculation was performed using the convolution/superposition algorithm. During planning, once the dose objectives of PTV_total_ and PTV_boost_ were achieved, the optimization was sustained to reduce the OAR dose until the plan could no longer be improved.

The ff-IMRT and hy-IMRT plans were created on the Eclipse Treatment Planning System (TPS version 13.6, Varian Medical Systems, Palo Alto, CA) for a Varian 23EX Linac. For ff-IMRT plans, the dose was contributed by five IMRT beams with the same gantry angle settings with the TD plans. In hy-IMRT plans, the same five IMRT beams and a pair of tangential CRT fields were used. The CRT fields delivered 70% of the *D*_p_ to the PTV_total_, and the other five IMRT fields were optimized based on the CRT beams, providing the remaining dose to PTV_total_ and PTV_boost_. Anisotropic analytical algorithm (AAA) and a grid of 2.5 × 2.5 mm^2^ were used for dose calculations.

### 2.3. Plan Evaluation

Multiple DVH metrics were extracted to compare the dose coverage of the target volumes and the dose sparing of OARs. For PTV_boost_, the conformity index (CI) was calculated using the formula CI = TV_PIV_^2^/(TV × PIV) [15], where TV_PIV_ is the target encompassed within the volume covered by the prescription isodose surface (PIV) and TV is the total volume of the target. The homogeneity index was calculated using HI = (*D*_2%_ − *D*_98%_)/*D*_p_. For PTV_total_, the quality of dose coverage (*Q*) [13] and the heterogeneity index (hI) [13] were calculated using *Q* = *D*_98%_/*D*_p_ and hI = *D*_2%_/*D*_98%_, respectively.

The NTCP for the lungs and heart and the SCCP for the lungs and contralateral breast were evaluated to quantify the risk of late injury of OARs. Prior to the calculation of radiobiological metrics, the physical dose was corrected for the fractionation effect using the linear-quadratic (LQ) model [16] (*α*/*β* = 3 Gy). The differential dose-volume histograms (dDVH) were then imported into an in-house MATLAB-based program (MathWorks, Natick, MA) to calculate the NTCP and SCCP values.

The NTCP for the lungs to developing pneumonitis grade ≥ 2 was estimated using the Lyman-Kutcher-Burman (LKB) model [17, 18]. The NTCP for the lungs was calculated with the following parameters: *D*_50_ = 24.5 Gy, *n* = 0.87, and *m* = 0.18 [19, 20].

The NTCP for cardiac mortality endpoint was calculated using the widely accepted relative seriality (RS) model [21]. The parameters used in the RS model were *D*_50_ = 52.3 Gy, *γ* = 1.28, and *s* = 1 [22].

The SCCP was calculated for the lungs and contralateral breast with the Schneider model [23, 24] using

(1) $$\begin{array}{c} {\text{SCCP}}_{\text{org}}={\text{In}}_{\text{org}}\cdot \underset{i}{\sum }\left(v_{i}\cdot D_{i}\cdot e^{-\alpha D_{j}}\right), \end{array}$$

where *α* is the cell radio sensitivity (Gy^−1^) and In_org_ is the absolute cancer incidence rate in percent per gray for the specific organ. The parameters used to calculate SCCP were *α* = 0.085 and In_org_ = 1.68%/Gy [23] for the lungs and *α* = 0.085 and In_org_ = 0.78%/Gy [24] for the contralateral breast, respectively.

To compare the delivery efficiency, the number of monitor units (MUs) and the delivery beam-on time were also analyzed.

### 2.4. Statistical Analysis

Statistics of the data were presented as mean ± standard deviation (SD). The differences of the dosimetric parameters and the radiobiological metrics between the five plans were analyzed using ANOVA and *post hoc* Tukey test with SPSS statistical software (version 23.0, Chicago, IL). The difference was considered statistically significant if *p* < 0.05.

## 3. Results

### 3.1. Planning Target Volumes

**Figure 1:**
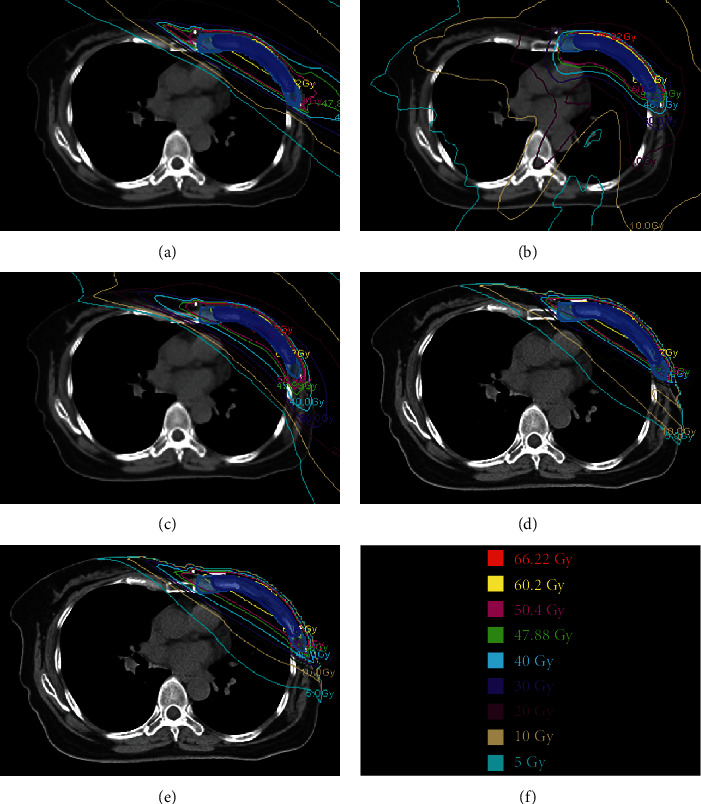
Isodose curves of one patient in axial view: (a) TD plan; (b) unb-HT plan; (c) b-HT plan; (d) ff-IMRT plan; (e) hy-IMRT plan; (f) isodose level.

**Figure 2:**
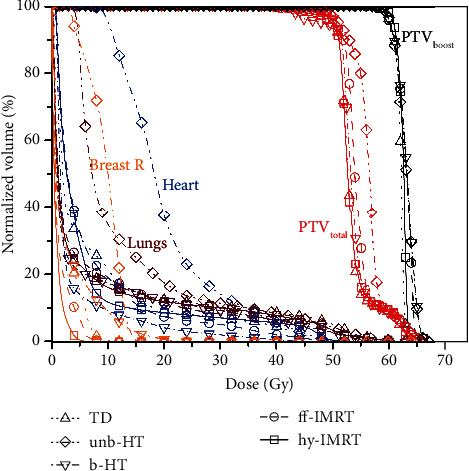
DVH for TD, unb-HT, b-HT, ff-IMRT, and hy-IMRT plans for a representative patient (PTV_boost_: black; PTV_total_: red; heart: blue; lungs: dark red; contralateral breast: orange).

The mean volume of PTV_boost_ and PTV_total_ of the twenty patients was 53.20 ± 22.28 cm^3^ (ranging from 22.7 to 113.1 cm^3^) and 609.9 ± 133.71 cm^3^ (ranging from 393.2 to 917.7 cm^3^), respectively. The dosimetric parameters, radiobiological metrics, and delivery efficiency results were summarized in Table 2. The *p* values for multiple comparisons using *post hoc* Tukey test are presented in Table 3. The isodose distributions and DVHs for one typical patient were shown in Figures 1 and 2, respectively.

All techniques meet the clinical requirements of PTV_boost_ and PTV_total_ dose coverage. For PTV_boost_, we found no significant difference in *D*_98%_, *D*_95%_, and *V*_110%_ (*p* > 0.05) among the five techniques, except that *D*_98%_ in unb-HT (59.49 ± 0.23 Gy) and in TD (59.45 ± 0.30 Gy) plans was slightly higher than that in ff-IMRT (59.22 ± 0.33 Gy). The hy-IMRT plans exhibited the lowest mean dose (61.67 ± 0.37 Gy) followed by TD plans (62.14 ± 0.53 Gy), while there was no significant difference between the other three techniques. The b-HT technique provides the optimal conformity (CI = 0.73 ± 0.08) followed by unb-HT (0.66 ± 0.07, *p* = 0.048), hy-IMRT (0.59 ± 0.10, *p* < 0.001), ff-IMRT (0.58 ± 0.10, *p* < 0.001), and TD (0.57 ± 0.09, *p* < 0.001). The best HI was achieved in hy-IMRT with 0.06 ± 0.01, followed by TD plans with 0.07 ± 0.02. Relatively lower HI values were provided by ff-IMRT (0.08 ± 0.02), unb-HT (0.09 ± 0.01), and b-HT (0.09 ± 0.02) plans, but there was no significant difference between them.

For PTV_total_, b-HT exhibited a lower *D*_98%_ (47.15 ± 2.58 Gy) than the other four techniques. There was no significant difference in *D*_95%_ of the five techniques. The unb-HT plans exhibited higher mean dose (55.36 ± 0.75 Gy) than the b-HT plans (54.09 ± 0.97 Gy, *p* < 0.001), the TD plans (53.78 ± 0.80 Gy, *p* < 0.001), the ff-IMRT plans (53.74 ± 0.82 Gy, *p* < 0.001), and the hy-IMRT plans (53.60 ± 0.56 Gy, *p* < 0.001). TD plans exhibited the best quality of dose coverage and homogeneity by showing the highest *Q* = 0.99 ± 0.02 and lowest hI = 1.26 ± 0.03, respectively. On the contrary, b-HT plans gave the lowest *Q* (0.94 ± 0.05, *p* < 0.001) and the highest hI (1.35 ± 0.09, *p* < 0.001).

### 3.2. Organs at Risk

For the dose sparing of the lungs, unb-HT plans had the highest *V*_5Gy_ and *V*_20Gy_ for the total lungs, *V*_5Gy_, *V*_20Gy_, and *D*_mean_ for the ipsilateral lung, and *V*_5Gy_ and *D*_mean_ for the contralateral lung, yielding the highest NTCP (0.97 ± 1.25‰) and SCCP (3.57 ± 0.05%) for the lungs among the five techniques. A statistically significant difference was found for the dose sparing of the lungs for unb-HT vs. other techniques (*p* < 0.001). For the other four techniques, b-HT exhibited the lowest NTCP (0.03 ± 0.03‰) and TD plans gave the lowest SCCP (1.25 ± 0.12%). TD plans exhibited the lowest *V*_5Gy_ (44.75 ± 3.19%) in the ipsilateral lung compared with hy-IMRT (47.85 ± 4.45%, *p* = 0.241), b-HT (49.83 ± 4.61%, *p* = 0.008), and ff-IMRT (50.59 ± 4.46%, *p* = 0.002), while hy-IMRT gave the lowest *V*_20Gy_ (24.56 ± 3.66%) and mean dose (13.84 ± 1.59 Gy) in the ipsilateral lung and the lowest *V*_5Gy_ (0.67 ± 0.66%) and mean dose (0.71 ± 0.23 Gy) in the contralateral lung, but there was no significant difference when compared with the other three techniques.

In terms of the dose to the heart, similarly, unb-HT plans delivered significantly higher *V*_5Gy_, *V*_10Gy_, *V*_20Gy_, *V*_30Gy_, and mean dose to the heart than the other techniques and induced a higher NTCP of 4.58 ± 3.62%, compared with the ff-IMRT (1.88 ± 2.32%, *p* < 0.001), hy-IMRT (2.06 ± 1.96%, *p* < 0.001), TD (2.05 ± 2.18%, *p* < 0.001), and b-HT (0.61 ± 0.73%, *p* < 0.001) plans. The b-HT plans achieved the lowest *V*_5Gy_, *V*_20Gy_, *V*_30Gy_, *D*_mean_, and NTCP, but no significant difference was found in these metrics when compared with the other techniques, except that *V*_5Gy_ in b-HT plans is significantly higher than that in ff-IMRT (18.20 ± 7.80% vs. 26.74 ± 9.82%, *p* = 0.012). The hy-IMRT and TD plans showed a better heart sparing in *V*_5Gy_, *V*_10Gy_, *V*_20Gy_, *V*_30Gy_, and *D*_mean_ than ff-IMRT, but these three techniques gave similar mean NTCP values for the heart.

Concerning the dose sparing of the contralateral breast, unb-HT plans revealed the highest mean dose of 9.86 ± 1.77 Gy and the highest SCCP of 2.75 ± 0.29%, followed by b-HT plans with a mean dose of 5.87 ± 2.36 Gy and SCCP of 1.65 ± 0.66%. Differences between the other three techniques were small and were not statistically significant.

### 3.3. Delivery Efficiency

The hy-IMRT plans and ff-IMRT plans showed similar delivery efficiency with lower MU values (993 ± 131 and 961 ± 150, respectively) and shorter beam-on time (99 ± 13 s and 96 ± 15 s, respectively) per fraction compared to the other three techniques. The b-HT plans had the highest number of MUs (12693 ± 1393, *p* < 0.001) and the longest beam-on time (880 ± 102 s, *p* < 0.001) among the five techniques.

## 4. Discussion

The present study evaluated five techniques for PMRT with SIB for breast cancer treatment. This is the first comprehensive and comparative evaluation of various modern techniques for PMRT with SIB. DVH indices were compared to assess the dose coverage for target volumes and the dose sparing for OARs. The value of NTCP and SCCP of OARs was calculated with previously published models to assess the late injury.

Overall, no one technique was uniformly superior for all criteria. All techniques provided acceptable dose coverage to the target volumes. Our analyses showed that TD and hy-IMRT could achieve a good balance of target dose coverage and dose sparing of OARs for PMRT with SIB treatment. The hy-IMRT and TD plans exhibited similar dose coverage, conformity, and homogeneity for target volumes and similar OAR sparing. TD plans slightly improved the dose coverage and heterogeneity for PTV_total_, but have longer delivery duration when compared with hy-IMRT plans. The ff-IMRT plans delivered a relatively higher lung and heart dose with similar dose coverage and homogeneity for the target volumes compared with TD and hy-IMRT plans. These three types of plans had better dose sparing for the contralateral breast when compared with the helical TomoTherapy plans, which can be attributed to the limited gantry angles in these three techniques.

We observed significantly worse dose sparing for the heart, lungs, and contralateral breast in unb-HT plans than that in other techniques. Moreover, the NTCP for the lungs and heart and the SCCP for the lungs and contralateral breast in unb-HT plans were the highest among all techniques. This is mainly because the beam direction is not limited enough due to the characteristics of the helical TomoTherapy (full arc helical beam delivery). Similar results have been reported by Xie et al. [10] for helical TomoTherapy for PMRT without SIB. Since the dose prescription in their study is different from that in the present study, a quantitative comparison is not practical. Although unb-HT plans can achieve good dose coverage to the target volumes, the clinical use of the unb-HT technique is somewhat less attractive for PMRT due to the potentially higher risk of radiation pneumonitis, cardiac disease, and second cancer.

Compared with the unb-HT plans, the b-HT plans significantly reduced the dose to the lungs and heart by employing the complete blocking structure to limit the freedom of beam angle. Note also for b-HT plans is the concave isodose curves just below the PTV_total_ (as is shown in Figure 1(a)). It indicates that the high dose volume in the lungs and heart could be compressed in b-HT plans. Patients with the proximity of the target volumes to the heart may benefit from the b-HT technique, whereas the clinical decision depends on the individual patient's plan. Moreover, b-HT plans had an inferior dose sparing of contralateral breast and may yield in a higher risk of second cancer compared with TD, ff-IMRT, and hy-IMRT plans. Further studies are required to further reduce the dose to the contralateral breast (especially for those young patients who experience long survival times) before implementing the b-HT technique for PMRT with SIB. Another disadvantage of b-HT plans is the significantly higher number of MU and longer treatment time (approximately 15 minutes on average) compared with other techniques. According to the clinical experience in our department, the daily treatment time for each patient should be shorter than 10 minutes, considering the patients' stability and the workload of the machine. The treatment time of b-HT plans could be shortened by using a field width of 5 cm instead of 2.5 cm. However, it needs further investigation to achieve a tradeoff between the treatment time and the plan quality. It is possible to further improve the plan quality in the b-HT mode by changing the machine parameters (pitch, field width, and MF) or optimization objectives. In the present study, the machine parameters were kept consistent for TD, b-HT, and unb-HT plans to not bias the results.

The biological dose-response models were used to compare various techniques in the current study. The age dependence effect was ignored, and only the solid tumor induction incidence was considered in the calculations of SCCP. Moreover, the influence of chemotherapy and other adjuvant treatment modalities to the risk of normal tissue complication and second cancer is not considered here. Hence, the NTCP and SCCP results presented in the present study are more appropriate to be used for relative comparisons between rival plans rather than for assessment of the absolute risk of the biological impact.

The deep-inspiration-breath-hold (DIBH) technique has been demonstrated to markedly improve the dose sparing of the heart and lungs during radiotherapy for breast cancer patients [25, 26]. However, treatment with free breathing is the standard procedure for PMRT patients in our institution, and hence, the DIBH technique was not considered in the current study. Actually, FB is the standard of care for breast cancer in most hospitals in China. This is a limitation of this study. Further studies are needed to investigate and compare various techniques with the DIBH technique for PMRT with SIB.

## 5. Conclusions

Five advanced PMRT techniques with SIB for the left breast cancer patients have been evaluated and compared in the present study. Our analyses support that the TD technique and hy-IMRT technique can provide a superior balance of target coverage, dose sparing of OARs, and delivery efficiency compared with the other three techniques.

## Figures and Tables

**Table 1:** Patient characteristics.

Characteristic	Value
Age (y)	
Median	49.5
Range	30-65
Menopausal status (*n*)	
Pre	12
Post	8
Histologic grading	
Grade 2	14
Grade 3	6
Tumor size (cm)	
Median	3.25
Range	1.5-10
ER/PR status (*n*)	
Negative	9
Positive	11
Her-2 status (*n*)	
Negative	14
Positive	6
Chemotherapy (*n*)	
Preoperative	13
Postoperative	7

**Table 2:** Summary of the dosimetric parameters, radiobiological indices, and delivery efficiency.

Parameter	TD	unb-HT	b-HT	ff-IMRT	hy-IMRT
PTV_boost_					
*D*_98%_ (Gy)	59.46 ± 0.22	59.49 ± 0.23	59.45 ± 0.30	59.22 ± 0.33	59.36 ± 0.28
*D*_95%_ (Gy)	60.22 ± 0.12	60.18 ± 0.15	60.23 ± 0.13	60.20 ± 0.00	60.20 ± 0.00
*D*_mean_ (Gy)	62.14 ± 0.53	62.66 ± 0.40	62.56 ± 0.70	62.48 ± 0.43	61.67 ± 0.37
*V*_110%_ (%)	0.04 ± 0.19	0.46 ± 0.72	0.43 ± 1.41	0.04 ± 0.18	0.00 ± 0.00
CI	0.57 ± 0.09	0.66 ± 0.07	0.73 ± 0.08	0.58 ± 0.10	0.59 ± 0.10
HI	0.07 ± 0.02	0.09 ± 0.01	0.09 ± 0.02	0.08 ± 0.02	0.06 ± 0.01
PTV_total_					
*D*_98%_ (Gy)	50.01 ± 0.80	48.78 ± 0.86	47.15 ± 2.58	48.55 ± 0.92	48.89 ± 0.95
*D*_95%_ (Gy)	50.67 ± 0.63	50.37 ± 0.75	50.33 ± 0.75	50.42 ± 0.18	50.43 ± 0.26
*D*_mean_ (Gy)	53.78 ± 0.80	55.36 ± 0.77	54.09 ± 0.97	53.74 ± 0.82	53.60 ± 0.56
*Q*	0.99 ± 0.02	0.97 ± 0.02	0.94 ± 0.05	0.96 ± 0.02	0.97 ± 0.02
hI	1.26 ± 0.03	1.31 ± 0.03	1.35 ± 0.09	1.31 ± 0.03	1.27 ± 0.03
Lungs					
*V*_5Gy_ (%)	20.28 ± 2.15	83.61 ± 7.98	22.75 ± 3.00	23.28 ± 2.75	20.93 ± 2.62
*V*_20Gy_ (%)	11.58 ± 1.43	15.94 ± 2.49	11.71 ± 1.31	11.31 ± 2.09	10.43 ± 1.69
NTCP (‰)	0.04 ± 0.03	0.97 ± 1.25	0.03 ± 0.03	0.06 ± 0.13	0.04 ± 0.04
SCCP (%)	1.25 ± 0.12	3.57 ± 0.05	1.44 ± 0.13	1.38 ± 0.15	1.27 ± 0.14
IL lung					
*V*_5Gy_ (%)	44.75 ± 3.19	83.46 ± 6.37	49.83 ± 4.61	50.59 ± 4.46	47.85 ± 4.45
*V*_20Gy_ (%)	26.52 ± 2.01	33.42 ± 3.29	27.10 ± 2.62	26.18 ± 4.25	24.56 ± 3.66
*D*_mean_ (Gy)	14.17 ± 1.28	18.01 ± 1.26	13.89 ± 1.36	14.16 ± 1.95	13.84 ± 1.59
CL lung					
*V*_5Gy_ (%)	1.71 ± 1.67	81.13 ± 12.60	2.28 ± 1.05	2.73 ± 1.93	0.67 ± 0.66
*D*_mean_ (Gy)	0.96 ± 0.25	8.43 ± 1.77	1.30 ± 0.91	0.92 ± 0.30	0.71 ± 0.23
Heart					
*V*_5Gy_ (%)	20.86 ± 8.76	99.89 ± 0.49	18.20 ± 7.80	26.74 ± 9.82	22.56 ± 9.99
*V*_10Gy_ (%)	15.31 ± 7.19	94.97 ± 3.93	12.49 ± 7.11	18.51 ± 7.84	11.82 ± 6.71
*V*_20Gy_ (%)	9.36 ± 5.10	55.70 ± 22.32	6.83 ± 5.18	9.98 ± 5.86	7.76 ± 5.34
*V*_30Gy_ (%)	6.20 ± 4.09	27.95 ± 20.16	3.51 ± 3.01	6.24 ± 5.06	6.08 ± 4.65
*D*_mean_ (Gy)	5.99 ± 2.58	27.95 ± 7.39	4.85 ± 2.04	6.65 ± 2.96	5.76 ± 2.77
NTCP (%)	2.05 ± 2.18	4.58 ± 3.62	0.61 ± 0.73	1.88 ± 2.32	2.06 ± 1.96
CL breast					
*D*_mean_ (Gy)	1.78 ± 1.21	9.86 ± 1.77	5.87 ± 2.36	1.51 ± 1.03	1.12 ± 1.09
SCCP (%)	0.63 ± 0.38	2.75 ± 0.29	1.65 ± 0.66	0.53 ± 0.31	0.34 ± 0.20
Spinal cord					
*D*_max_ (Gy)	32.25 ± 8.63	37.41 ± 2.93	33.18 ± 6.26	30.77 ± 9.41	30.87 ± 9.41
Delivery efficiency					
MU	5514 ± 634	7088 ± 499	12693 ± 1393	961 ± 150	993 ± 131
Time (s)	434 ± 44	501 ± 34	880 ± 102	96 ± 15	99 ± 13

TD: TomoDirect; unb-HT: helical TomoTherapy without blocking structure; b-HT: helical TomoTherapy with blocking structure; ff-IMRT: fixed-field IMRT; hy-IMRT: hybrid IMRT. *D*_*x*_ (Gy): dose absorbed by certain percentage (%) of the structure; *V*_*x*_ (%): fractional volume exposed to certain radiation dose; IL lung: ipsilateral lung; CL lung: contralateral lung; CL breast: contralateral breast; similarly hereinafter.

**Table 3:** *p* values for multiple comparisons using *post hoc* Tukey test.

Parameter	TD vs. unb-HT	TD vs. b-HT	TD vs. ff-IMRT	TD vs. hy-IMRT	unb-HT vs. b-HT	unb-HT vs. ff-IMRT	unb-HT vs. hy-IMRT	b-HT vs. ff-IMRT	b-HT vs. hy-MRT	ff-IMRT vs. hy-IMRT
PTV_boost_										
*D*_98%_	0.997	1.000	0.039^∗^	0.771	0.989	0.015^∗^	0.562	0.056	0.843	0.435
*D*_95%_	0.773	0.999	0.985	0.985	0.616	0.968	0.968	0.9360	0.936	1.000
*D*_mean_	0.012^∗^	0.068	0.201	0.030^∗^	0.972	0.801	<0.001^∗^	0.988	<0.001^∗^	<0.001^∗^
*V*_110%_	1.000	0.452	1.000	1.000	1.000	0.361	0.270	0.442	0.340	1.000
CI	0.019^∗^	<0.001^∗^	0.989	0.976	0.048^∗^	0.069	0.090	<0.001^∗^	<0.001^∗^	1.000
HI	<0.001^∗^	0.005^∗^	0.040^∗^	0.898	0.720	0.288	<0.001^∗^	0.952	<0.001^∗^	0.003^∗^
PTV_total_										
*D*_98%_	0.050	<0.001^∗^	0.012^∗^	0.094	0.003^∗^	0.985	1.000	0.018^∗^	0.001^∗^	1.000
*D*_95%_	0.468	0.344	0.643	0.698	1.000	0.999	0.997	.0.988	0.981	1.000
*D*_mean_	<0.001^∗^	0.733	1.000	0.952	<0.001^∗^	<0.001^∗^	<0.001^∗^	0.643	0.299	0.979
*Q*	0.061	<0.001^∗^	0.021^∗^	0.119	0.005^∗^	0.995	0.999	0.018^∗^	0.002^∗^	0.961
hI	0.016^∗^	<0.001^∗^	0.016^∗^	0.846	0.024^∗^	1.000	0.201	0.024^∗^	<0.001^∗^	0.201
Lungs										
*V*_5Gy_	<0.001^∗^	0.364	0.183	0.989	<0.001^∗^	<0.001^∗^	<0.001^∗^	0.995	0.664	0.418
*V*_20Gy_	<0.001^∗^	0.999	0.991	0.295	<0.001^∗^	<0.001^∗^	<0.001^∗^	0.959	0.192	0.562
NTCP	<0.001^∗^	1.000	1.000	1.000	<0.001^∗^	<0.001^∗^	<0.001^∗^	1.000	1.000	1.000
SCCP	<0.001^∗^	0.130	0.485	1.000	<0.001^∗^	<0.001^∗^	<0.001^∗^	0.942	0.194	0.610
IL lung										
*V*_5Gy_	<0.001^∗^	0.008^∗^	0.002^∗^	0.241	<0.001^∗^	<0.001^∗^	<0.001^∗^	0.987	0.673	0.361
*V*_20Gy_	<0.001^∗^	0.980	0.998	0.327	<0.001^∗^	<0.001^∗^	<0.001^∗^	0.900	0.109	0.520
*D*_mean_	<0.001^∗^	0.977	1.000	0.956	<0.001^∗^	<0.001^∗^	<0.001^∗^	0.979	1.000	0.959
CL lung										
*V*_5Gy_	<0.001^∗^	0.998	0.984	0.977	<0.001^∗^	<0.001^∗^	<0.001^∗^	1.000	0.891	0.796
*D*_mean_	<0.001^∗^	0.997	1.000	0.860	<0.001^∗^	<0.001^∗^	<0.001^∗^	0.986	0.678	0.927
Heart										
*V*_5Gy_	<0.001^∗^	0.843	0.161	0.965	<0.001^∗^	<0.001^∗^	<0.001^∗^	0.012^∗^	0.449	0.489
*V*_10Gy_	<0.001^∗^	0.674	0.556	0.471	<0.001^∗^	<0.001^∗^	<0.001^∗^	0.043^∗^	0.998	0.018^∗^
*V*_20Gy_	<0.001^∗^	0.956	1.000	0.992	<0.001^∗^	<0.001^∗^	<0.001^∗^	0.907	0.999	0.973
*V*_30Gy_	<0.001^∗^	0.908	1.000	1.000	<0.001^∗^	<0.001^∗^	<0.001^∗^	0.903	0.920	1.000
*D*_mean_	<0.001^∗^	0.901	0.986	1.000	<0.001^∗^	<0.001^∗^	<0.001^∗^	0.630	0.954	0.958
NTCP	0.008^∗^	0.303	0.999	1.000	<0.004^∗^	<0.001^∗^	<0.009^∗^	0.430	0.297	0.999
CL breast										
*D*_mean_	<0.001^∗^	<0.001^∗^	0.983	0.682	<0.001^∗^	<0.001^∗^	<0.001^∗^	0.001^∗^	<0.001^∗^	0.936
SCCP	<0.001^∗^	<0.001^∗^	0.931	0.152	<0.001^∗^	<0.001^∗^	<0.001^∗^	0.001^∗^	<0.001^∗^	0.559
Spinal cord										
*D*_max_	0.225	0.995	0.975	0.980	0.423	0.060	0.066	0.862	0.879	1.000
Delivery efficiency										
MU	<0.001^∗^	<0.001^∗^	<0.001^∗^	<0.001^∗^	<0.001^∗^	<0.001^∗^	<0.001^∗^	<0.001^∗^	<0.001^∗^	1.000
Time	0.001^∗^	<0.001^∗^	<0.001^∗^	<0.001^∗^	<0.001^∗^	<0.001^∗^	<0.001^∗^	<0.001^∗^	<0.001^∗^	1.000

∗The difference is statistically significant.

## Data Availability

The raw data extracted from the dose-volume-histogram results for each treatment plan, the raw data of statistics analysis, and the MATLAB code used in this study are available from the corresponding author by request.

## References

[1] Ragaz J., Jackson S. M., Le N. (1997). Adjuvant radiotherapy and chemotherapy in node-positive premenopausal women with breast cancer. *The New England Journal of Medicine*.

[2] Overgaard M., Hansen P. S., Overgaard J. (1997). Postoperative radiotherapy in high-risk premenopausal women with breast cancer who receive adjuvant chemotherapy. *The New England Journal of Medicine*.

[3] Panoff J. E., Takita C., Hurley J. (2012). Higher chest wall dose results in improved locoregional outcome in patients receiving postmastectomy radiation. *International Journal of Radiation Oncology·Biology·Physics*.

[4] Blitzblau R. C., Horton J. K. (2013). Treatment planning technique in patients receiving postmastectomy radiation therapy. *Practical Radiation Oncology*.

[5] Huang E.-Y., Chen H.-C., Sun L.-M. (2006). Multivariate analyses of locoregional recurrences and skin complications after postmastectomy radiotherapy using electrons or photons. *International Journal of Radiation Oncology·Biology·Physics*.

[6] Chung M. J., Kim S. H., Lee J. H., Suh Y. J. (2015). A dosimetric comparative analysis of TomoDirect and three-dimensional conformal radiotherapy in early breast cancer. *Journal of Breast Cancer*.

[7] Hashimoto H., Omura M., Matsui K. (2015). Tangent field technique of TomoDirect improves dose distribution for whole-breast irradiation. *Journal of Applied Clinical Medical Physics*.

[8] Fields E. C., Rabinovitch R., Ryan N. E., Miften M., Westerly D. C. (2013). A detailed evaluation of TomoDirect 3DCRT planning for whole-breast radiation therapy. *Medical Dosimetry*.

[9] Balaji K., Yadav P., BalajiSubramanian S., Radha C. A., Ramasubramanian V. (2018). Hybrid volumetric modulated arc therapy for chest wall irradiation: for a good plan, get the right mixture. *Physica Medica*.

[10] Xie Y., Bourgeois D., Guo B., Zhang R. (2020). Postmastectomy radiotherapy for left-sided breast cancer patients: comparison of advanced techniques. *Medical Dosimetry*.

[11] Rong Y., Yadav P., Welsh J. S., Fahner T., Paliwal B. (2012). Postmastectomy radiotherapy with integrated scar boost using helical tomotherapy. *Medical Dosimetry*.

[12] Parijs H. V., Reynders T., Heuninckx K., Verellen D., Storme G., De Ridder M. (2014). Breast conserving treatment for breast cancer: dosimetric comparison of different non-invasive techniques for additional boost delivery. *Radiation Oncology*.

[13] Aly M. M. O. M., Glatting G., Jahnke L., Wenz F., Abo-Madyan Y. (2015). Comparison of breast simultaneous integrated boost (SIB) radiotherapy techniques. *Radiation Oncology*.

[14] Franco P., Catuzzo P., Cante D. (2018). TomoDirect: an efficient means to deliver radiation at static angles with tomotherapy. *Tumori*.

[15] Paddick I. (2000). A simple scoring ratio to index the conformity of radiosurgical treatment plans. *Journal of Neurosurgery*.

[16] Lebesque J. V., Keus R. B. (1991). The simultaneous boost technique: the concept of relative normalized total dose. *Radiotherapy and Oncology*.

[17] Lyman J. (1992). Normal tissue complication probabilities: variable dose per fraction. *International Journal of Radiation Oncology·Biology·Physics*.

[18] Kutcher G. J., Burman C. (1989). Calculation of complication probability factors for non-uniform normal tissue irradiation: the effective volume method gerald. *International Journal of Radiation Oncology·Biology·Physics*.

[19] Burman C., Kutcher G. J., Emami B., Goitein M. (1991). Fitting of normal tissue tolerance data to an analytic function. *International Journal of Radiation Oncology·Biology·Physics*.

[20] Emami B., Lyman J., Brown A. (1991). Tolerance of normal tissue to therapeutic irradiation. *International Journal of Radiation Oncology·Biology·Physics*.

[21] Källman P., Ågren A., Brahme A. (1992). Tumour and normal tissue responses to fractionated non-uniform dose delivery. *International Journal of Radiation Biology*.

[22] Gagliardi G., Lax I., Ottolenghi A., Rutqvist L. E. (1996). Long-term cardiac mortality after radiotherapy of breast cancer--application of the relative seriality model. *The British Journal of Radiology*.

[23] Schneider U., Kaser-Hotz B. (2005). Radiation risk estimates after radiotherapy: application of the organ equivalent dose concept to plateau dose-response relationships. *Radiation and Environmental Biophysics*.

[24] Schneider U., Kaser-Hotz B. (2005). A simple dose-response relationship for modeling secondary cancer incidence after radiotherapy. *Zeitschrift für Medizinische Physik*.

[25] Pandeli C., Smyth L. M., David S., See A. W. (2019). Dose reduction to organs at risk with deep-inspiration breath-hold during right breast radiotherapy: a treatment planning study. *Radiation Oncology*.

[26] Bruzzaniti V., Abate A., Pinnarò P. (2013). Dosimetric and clinical advantages of deep inspiration breath-hold (DIBH) during radiotherapy of breast cancer. *Journal of Experimental & Clinical Cancer Research*.

